# A Case of Gemella morbillorum Causing Multi-valvular Endocarditis

**DOI:** 10.7759/cureus.53716

**Published:** 2024-02-06

**Authors:** Gabriel Panama, Adolfo Martinez, Majid Yavari, Andrew Geunwon Kim, George Abela

**Affiliations:** 1 Department of Internal Medicine, Michigan State University, East Lansing, USA; 2 Department of Cardiology, Michigan State University, East Lansing, USA

**Keywords:** infective endocarditis, mitral valve insufficiency, aortic valve insufficiency, gemella endocarditis, gemella

## Abstract

This is the case of a 31-year-old man with no significant past medical history who presented to the emergency department experiencing persistent fevers, chills, and malaise for the past 2-3 weeks. During this period, he had multiple urgent care visits for possible left-sided otitis media which was treated with short a course of Augmentin. While on antibiotics his symptoms would improve, but they would reappear once he had finished treatment. The patient also had significant dental carries with a chronic right molar infection. At the emergency department, blood cultures grew two out of two* Gemella morbillorum*. Transthoracic echocardiography showed a 1 cm x 0.5 cm mobile density on the left coronary cusp of the aortic valve with moderate-severe aortic insufficiency. The patient was started on empiric IV vancomycin. Further workup revealed that the source of infection was dental carries. While proceeding with a transesophageal echocardiogram, the patient went into flash pulmonary edema requiring ICU admission. Imaging revealed an elongated 1.7 cm x 0.6 cm vegetation attached to the base of the left coronary cusp on the left ventricular outflow tract side with severe aortic regurgitation and a small 0.8 cm x 0.8 cm vegetation on the atrial side of the anterior mitral leaflet at A2 associated with mitral leaflet perforation with severe mitral regurgitation. Oral surgery removed the infected teeth. Cardiothoracic surgery performed open heart valve replacement which revealed a completely destroyed aortic valve, droplet vegetation, and destruction of the mitral valve leading to mechanical valve replacement. The patient received a two-week course of gentamycin while in the ICU with meropenem. Once sensitivities were back, he was switched to IV penicillin therapy for a total of six weeks.

## Introduction

Infective endocarditis (IE) is a disease that if left untreated has a high mortality and morbidity. With the increasing use of intravenous (IV) lines, implants, and cardiac devices, the etiology of IE in developed countries has changed. *Staphylococcus aureus* is now the most prevalent microorganism, followed by* Streptococcus viridans group.* These account for approximately 80-90% of all cases of endocarditis [1]. Other organisms include the HACEK group (*Haemophilus parainfluenzae*, *Aggregatibacter *spp., *Cardiobacterium *spp., *Eikenella corrodens*, *Kingella *spp.). These are gram-negative bacteria part of the oral flora and account for 1-3% of cases [2].

*Gemella morbillorum* is a gram positive, catalase negative, facultatively anerobic coccus found in the genitourinary and gastrointestinal flora. It is a rare cause of IE, with 24 cases described up until 2010 [3]. A total of 16 cases have been reported between 2010 and 2021 [4].

Predisposing factors for IE include IV drug use, valvular disease, prosthetic valves, intravascular devices, and congenital heart disease. The diagnosis of IE is established with the modified Duke criteria, which has a sensitivity of 80% [1]. Transthoracic echocardiography (TEE) is used as an initial imaging modality to assess for vegetation and valvular abnormalities. It is often followed by TEE to confirm the diagnosis. Some known complications of endocarditis include heart failure, valvular abnormalities, and embolic phenomena, with the brain being the most common site of embolization. Management varies depending on the case presentation. However, complicated cases often require a multi-disciplinary approach.

## Case presentation

This is the case of a 31-year-old man with a history of poor oral hygiene and a recent right molar teeth infection who presented to the emergency department complaining of 3-4 weeks of fever, chills, and malaise. He was seen initially twice at urgent care where he was diagnosed with otitis media and Augmentin was prescribed. Whilst on antibiotics, his symptoms would subside, but these would recur once he finished the antibiotic course. Due to persistent symptoms, he visited the emergency department where two sets of blood cultures grew *G. morbillorum*. A TTE showed a 1 cm x 0.5 cm mobile echodensity on the left coronary cusp of the aortic valve and moderate to severe aortic regurgitation. Given these findings, he was started empirically on vancomycin and he was transferred to a tertiary center for evaluation by cardiothoracic surgery.

On arrival at our center, vital signs were within normal limits. Physical examination was remarkable for a systolic murmur. No vascular, immunologic, or embolic signs were noted. Initial labs were remarkable for a CRP 9.5 mg/dl (normal range < 1.0 mg/dl) and ESR 75 mm/hr (normal range 0-20 mm/hr), WBC. Antibiotics were switched to penicillin, gentamicin, and metronidazole.

Whilst undergoing TEE the patient became hypoxic and was placed on bilevel positive airway pressure (BIPAP). He was subsequently transferred to the ICU for further monitoring. A CT scan of the chest showed flash pulmonary edema. He was diuresed and eventually weaned off BIPAP. A TEE showed severe aortic regurgitation (Videos 1, 2) and mitral regurgitation. There was a small 0.8 cm x 0.8 cm vegetation on the atrial side of the anterior mitral leaflet at A2 associated with mitral leaflet perforation (Videos 3, 4). Prior to valve replacement, the oral surgery team performed teeth extraction and debridement of a vestibular abscess. Subsequently, the patient underwent open heart surgery for mechanical aortic and mitral valve replacement. There was visible destruction of the valves on gross examination, including perforation of the anterior leaflet of the mitral valve. Valve cultures showed were negative.

**Video 1:** TEE of aortic valve Long 1.7 cm x 0.6 cm vegetation attached to the base of the left coronary cusp on the left ventricular outflow side. TEE: Transthoracic echocardiography

**Video 2:** TEE with color flow Doppler of the aortic valve Severe aortic regurgitation was noted. TEE: Transthoracic echocardiography

**Video 3:** TEE with two chamber views focused on the mitral valve There is a 0.8 cm x 0.8 cm vegetation on the atrial side of the anterior mitral valve leaflet at A2 associated with mitral leaflet perforation. TEE: Transthoracic echocardiography

**Video 4:** TEE with color flow Doppler taking a closer look at the mitral valve perforation TEE: Transthoracic echocardiography

The post-op period was complicated by vasoplegia after surgery, for which the patient required three vasopressors for a period of time. He also developed a new fever along with worsening leukocytosis. Septic shock was considered amongst the differential diagnoses and antibiotics were switched to meropenem and gentamycin. The ICU stay is complicated with circulatory shock leading to acute kidney injury requiring continuous renal replacement therapy (CRRT) and ischemic hepatitis.

Initial blood culture sensitivities demonstrated that the bacteria was pan-sensitive to all antibiotics, for which the antibiotic regimen was switched to penicillin 4 million units IV to complete an antibiotic course of six weeks.

After a one-week stay in the ICU, the patient was weaned off vasopressors and CRRT. He was discharged to a long-term acute care hospital to continue his care.

## Discussion

*Gemella *spp. are anaerobic gram-positive cocci found in the oral cavity, gastrointestinal, and genitourinary tract of humans. Predisposing risk factors for Gemella endocarditis include poor oral dentition, dental surgery, and congenital heart disease [4]. These are known common risk factors for IE independent of the etiology. They also correlate with Gemella’s natural habitat within the human body.

A systemic review of 83 cases identified five Gemella species as causative agents of IE.* G. morbillorum, Gemella haemolysans*, *Gemella sanguinis, Gemella bergeriae, *and *Gemella taiwanesis*. *Morbillorum *and *haemolysans *were the most common. The aortic and mitral valves were the most commonly affected [5]. The most common form of presentation was subacute IE with days to weeks of fatigue and malaise [3,4,6]. Our case presented as a combination of subacute symptoms along with severe valvular disease.

*Gemella *spp. can be difficult to identify. Bacterial cells can sometimes appear elongated and for this reason, it was first described as a *Neisseria*. It can decolorize during gram stain making it appear as a gram variable or negative. If blood cultures are negative and there is a high clinical suspicion, polymerase chase reaction (PCR) of the 16S rRNA gene can help identify Gemella [7]. Valve biopsy is the gold standard for making a histologic diagnosis, valve cultures tend to have a high bacterial contamination and thus are not reliable. Cultures have a negative predictive value of 56%, a sensitivity of 15%, and a specificity of 13% [2]. Valve tissue PCR is preferred over whole blood PCR in cases where blood cultures are non-diagnostic. Due to the complexity of the diagnosis, the modified Duke criteria incorporate clinical, microbiological, and echocardiographic findings to diagnose IE. By doing so, it has a sensitivity of 80% in native valve endocarditis [8].

TTE is used as a first-line imaging modality. It has a sensitivity of 70% in native valves and 50% in prosthetic valves. TEE is better at assessing valvular structures. TEE should be done in cases with* S. aureus* bacteremia, prosthetic valves, intracardiac devices, non-diagnostic TTE, and positive TTE [8].

The European Society of Cardiology guidelines suggest empirical antibiotics should cover *Staphylococcus*, *Streptococcus*, and *Enterococcus*. In cases of healthcare-associated IE coverage for methicillin-resistant staphylococci is warranted. Proposed empiric regimens include ampicillin 12 g/day IV in 4-6 doses and vancomycin 30 mg/kg/day IV in two doses [8]. *Gemella *spp. was found to be susceptible to penicillin, vancomycin, cephalosporins, macrolides, and aminoglycosides [6,9]. Thus, in most cases, empiric treatment should cover Gemella until results from blood cultures allow it to be tailored for specific antibiotics.

## Conclusions

IE has a high mortality and morbidity if left untreated.* G. morbillorum *is a bacteria part of the human oral flora that has been found to cause a few cases of IE. Although it is a pathogen with low virulence, it should be a bacteria to bear in mind, particularly in cases that present as subacute endocarditis with poor oral hygiene. Previous case reports and studies have shown that most cases of Gemella are susceptible to most antibiotic regimens, thus it should be covered despite being rare.
